# Carriage of two carbapenem-resistance genes in *Pseudomonas aeruginosa* isolated from hospital-acquired infections in children from Costa Rica: the importance of local epidemiology

**DOI:** 10.1186/s13756-021-00942-7

**Published:** 2021-04-28

**Authors:** Cristian Pérez-Corrales, Valeria Peralta-Barquero, Christopher Mairena-Acuña

**Affiliations:** División de Microbiología, Laboratorio Clínico, Hospital Nacional de Niños “Dr. Carlos Sáenz Herrera”, Centro de Ciencias Médicas de La Caja Costarricense del Seguro Social, PO Box: 1654-1000, San José, Costa Rica

**Keywords:** Carbapenem-Resistant *Pseudomonas aeruginosa*, Hospital-acquired infections

## Abstract

**Background:**

The assessment of Hospital-acquired infections due to multidrug-resistant bacteria involves the use of a variety of commercial and laboratory-developed tests to detect antimicrobial resistance genes in bacterial pathogens; however, few are evaluated for use in low- and middle-income countries.

**Methods:**

We used whole-genome sequencing, rapid commercial molecular tests, laboratory-developed tests and routine culture testing.

**Results:**

We identified the carriage of the metallo-β-lactamase *bla*_VIM-2_ and *bla*_IMP-18_ alleles in Carbapenem-Resistant *Pseudomonas aeruginosa* infections among children in Costa Rica.

**Conclusions:**

The *bla*_IMP-18_ allele is not present in the most frequently used commercial tests; thus, it is possible that the circulation of this resistance gene may be underdiagnosed in Costa Rica.

## Background

The global increase of infections due to multidrug-resistant bacteria remains a public health and sustainable development problem [1]. The global antimicrobial resistance surveillance system (GLASS) encourages healthcare authorities to increase laboratory capacity. To improve surveillance and diagnostic stewardship, GLASS also recommends the implementation of rapid, accurate diagnostic testing for antibiotic resistance [2]. The methods for the detection of resistance mechanisms are diverse, ranging from phenotypic to more complex genotypic tests, and whole-genome sequencing (WGS). The continuous development of diagnostic tests in and for high-income countries makes them available for other countries at a good price. However, information on antimicrobial resistance in low- and middle-income countries is scarce, as is the performance of these kits in different epidemiological contexts. Carbapenem-Resistant *Pseudomonas aeruginosa* (CRPA) is frequently isolated from healthcare-associated infections (HAI). The mechanisms of CRPA resistance can be driven by porin loss, efflux pump activity, or by horizontal gene transfer encoded in plasmids [3]. In Costa Rica, the National Children’s Hospital is a tertiary referral hospital within the socialized medical care system. HAI assessment has recently improved in this hospital with the implementation of a diagnostic stewardship program, which was established according to its needs and resources. To better understand CRPA circulation in Costa Rica and compare detection methods, we studied *P. aeruginosa* isolated from pediatric patients with HAI between late 2018 and 2020 using conventional phenotypic methods, rapid molecular test, and WGS.

## Methods

Samples from patients with suspected HAI were collected and transported to the laboratory following the general specimen collection and transport techniques [4]. A total of 32 *P. aeruginosa* isolates were analyzed using all of the following methods: conventional phenotypic methods, molecular tests, and whole-genome sequencing. Identification and susceptibility to antimicrobials were performed by automated Vitek-2 Systems (BioMérieux, Marcy-l’Étoile, France) using CLSI breakpoints for Carbapenems (MIC ≥ 8 µg/mL) [5]. Phenotypic studies included modified carbapenem inhibition test (mCIM) according to CLSI [5] and metallo-ß-lactamase (MBL) E-test (BioMérieux, Marcy-l’Étoile, France). Molecular analyses for detection of genes conferring resistance included Xpert Carba Test (Cepheid, Sunnyvale CA, USA) for detection of genes encoding carbapenemases KPC, VIM, IMP, NDM, OXA-48; AMR Flow-Chip hybridization (Master Diagnóstica, Granada, Spain) for detection of genes encoding extended-spectrum beta-lactamases SHV, CTX-M; class A carbapenemases GES, SME, KPC, IMI; class B carbapenemases SIM, GIM, SPM, NDM, VIM, IMP and class D carbapenemases OXA23-like, 24-like, 48-like, 51-like and 58-like; and detection of genes encoding carbapenemases IMP and VIM by Polimerase Chain Reaction (PCR) Laboratory-Developed Tests (LDT) using primers described elsewhere [6]. When required, DNA was extracted using MagNA Pure (Roche Diagnostics, Basel, Switzerland), quantified by Quantus (Promega, Madison WI, USA), and verified by Qiaxcell (QIAgen, Germantown MD, USA). The whole genome was prepared using the Nextera Flex library preparation standard protocol and paired-end fragment (151 bp) sequencing (MiSeq, Illumina Inc., San Diego CA, USA). A general pipeline for read quality (FastQC) [7], trimming (Trimmomatic) [8], genome assembly (Shovill) [9], genome annotation (Prokka) [10] and resistance-gene finder (ABRicate) [11] was performed using Galaxy Community Hub [12].

## Results

A total of 8 out of the 32 bacterial isolates identified as *P. aeruginosa* exhibited resistance to imipenem and meropenem by automated means. In all 8 strains, mCIM was positive, indicating carbapenemase activity. Furthermore, MBL E-test methods confirmed the carbapenem resistance due to the presence of metallo-β-lactamase in all strains. The molecular methods classified all 8 strains in agreement with the observed phenotypic assays. However, GenXpert Carba RT-PCR, as well as the reverse-hybridization assay showed positive results for *bla*_VIM_ detection only. Conventional PCR using LDT detected both *bla*_VIM_ and *bla*_IMP_ genes. The analyses of resistance genes using the WGS data (ABRicate) [11] identified alleles *bla*_VIM-2_ and *bla*_IMP-18_. Taken together, 4 out of the 8 strains exhibited both *bla*_VIM-2_ and *bla*_IMP-18_ alleles. The remaining 4 strains harbored only the *bla*_VIM-2_ gene.

## Discussion

Carbapenems constitute one of the final lines of defense against resistant bacteria, particularly Gram-negative bacilli. CRPA is ranked as “critical priority” for research and development for new drugs and to implement antimicrobial stewardship initiatives by the World Health Organization [13]. Detecting the mechanisms of resistance to antimicrobials provide a helpful tool to prevent and control HAIs, especially considering the growing evidence of mobile genetic elements mediating and exacerbating nosocomial outbreaks [14, 15]. To address these critical pathogens, we implemented commercial rapid molecular tests, hybridization assays, and whole-genome sequencing to analyze CRPA in HAI and compare the aforementioned methods.

Eight of the 32 *P. aeruginosa* isolates from HAI in the National Children’s Hospital of Costa Rica exhibited carbapenem resistance. The presence of the *bla*_VIM_ gene was identified using two different commercial kits. However, when testing the same isolates by PCR LDT, we also detected the *bla*_IMP_ gene in addition to the *bla*_VIM_ gene. WGS analyses confirmed the presence of alleles *bla*_VIM-2_ and *bla*_IMP-18_, which were previously described in one of the main hospitals treating adults in the country [16]. Both genes are known to encode metallo-β-lactamase that confer resistance to carbapenems. Our analyses provide valuable information about the circulation of *P. aeruginosa* carrying *bla*_IMP-18_ and *bla*_VIM-2_ alleles in pediatric infections in Costa Rica. Moreover, we identified that allele *bla*_IMP-18_ is present in HAI, but not targeted in the current rapid molecular technologies available in Costa Rica. This could lead to miss-identification of the *P. aeruginosa* resistance mechanisms if healthcare facilities don’t have access to redundant methods for confirmation.

This work brings together the context of hospital-acquired infections, antimicrobial stewardship, diagnostic stewardship, availability of diagnostic methods, and whole genome sequencing to highlight the importance of local epidemiology when adopting strategies to fight antimicrobial resistance. Similar situations can be observed globally as infections due to multidrug-resistant bacteria increase and new technologies become available. Rapid molecular tests for the detection of antibacterial resistance continue to expand around the world. The availability, presentation, multiplex format, international validation, and low price makes these kits very attractive to use in economically constrained countries. However, they are developed for high-income countries in accordance with their needs and epidemiological contexts; thus, they might not have all of the targets that are circulating in other countries.

## Conclusions

The results of this study highlight the importance of knowing the local epidemiology when monitoring for CRPA in the context of HAIs using rapid molecular tests that were originally created for a different country.

## Data Availability

The datasets used and/or analysed during the current study are available from the corresponding author on reasonable request.

## Abbreviations

GLASS: The global antimicrobial resistance surveillance system
WGS: Whole-genome sequencing
CRPA: Carbapenem-resistant *Pseudomonas aeruginosa*
HAI: Hospital-acquired infections
CLSI: Clinical Laboratory Standards Institute
mCIM: Modified carbapenem inhibition test
LDT: Laboratory-developed tests
MBL: Metallo-ß-lactamase
PCR: Polimerase chain reaction
